# Evaluating the impact of clinical librarians on clinical questions during inpatient rounds

**DOI:** 10.5195/jmla.2018.254

**Published:** 2018-04-01

**Authors:** Riley Brian, Nicola Orlov, Debra Werner, Shannon K. Martin, Vineet M. Arora, Maria Alkureishi

**Affiliations:** Medical Student, University of Chicago Pritzker School of Medicine, Chicago, IL; Assistant Professor of Pediatrics, Department of Academic Pediatrics, University of Chicago, Chicago, IL; Librarian for Science Instruction and Outreach and Biomedical Reference, John Crerar Library, University of Chicago, Chicago, IL; Assistant Professor of Medicine, Department of Medicine, University of Chicago, Chicago, IL; Assistant Professor of Medicine, Department of Medicine, University of Chicago, Chicago, IL; Assistant Professor of Pediatrics, Department of Academic Pediatrics, University of Chicago, Chicago, IL

## Abstract

**Objective:**

The investigation sought to determine the effects of a clinical librarian (CL) on inpatient team clinical questioning quality and quantity, learner self-reported literature searching skills, and use of evidence-based medicine (EBM).

**Methods:**

Clinical questioning was observed over 50 days of inpatient pediatric and internal medicine attending rounds. A CL was present for 25 days and absent for 25 days. Questioning was compared between groups. Question quality was assessed by a blinded evaluator, who used a rubric adapted from the Fresno Test of Competence in Evidence-Based Medicine. Team members were surveyed to assess perceived impacts of the CL on rounds.

**Results:**

Rounds with a CL (CLR) were associated with significantly increased median number of questions asked (5 questions CLR vs. 3 NCLR; *p*<0.01) and answered (3 CLR vs. 2 NCLR; *p*<0.01) compared to rounds without a CL (NCLR). CLR were also associated with increased mean time spent asking (1.39 minutes CLR vs. 0.52 NCLR; *p*<0.01) and answering (2.15 minutes CLR vs. 1.05 NCLR; *p*=0.02) questions. Rounding time per patient was not significantly different between CLR and NCLR. Questions during CLR were 2 times higher in adapted Fresno Test quality than during NCLR (*p*<0.01). Select participants described how the CL’s presence improved their EBM skills and care decisions.

**Conclusions:**

Inpatient CLR were associated with more and improved clinical questioning and subjectively perceived to improve clinicians’ EBM skills. CLs may directly affect patient care; further study is required to assess this. CLs on inpatient rounds may be an effective means for clinicians to learn and use EBM skills.

## INTRODUCTION

The increasing volume of complex medical literature and concurrent time constraints have hindered clinicians’ abilities to search for information and apply evidence-based medicine (EBM) skills to clinical practice [1–4]. In fact, studies have shown that clinicians do not pursue or find answers for about half of their clinical questions that arise during everyday practice [5–8]. The greatest barrier to answering clinical questions, insufficient time, has also contributed to physician burnout and poor patient outcomes [9, 10]. Indeed, time pressure may detract from physicians’ satisfaction and can stress their relationships with patients [10, 11].

Clinical librarians (CLs), also known as medical or hospital librarians, have sought to address this problem and have been increasingly involved in clinical practice since the 1970s [12]. Since that time, a growing focus on lifelong learning by the American Association of Medical Colleges (AAMC) and the Accreditation Council for Graduate Medical Education (ACGME) has kindled discussion about the potential role of CLs in medical education [12–14]. When they are involved in clinical practice and medical education, CLs can have positive effects in multiple capacities by planning curricula, facilitating journal clubs, and training clinicians in the use of information services [2, 15–21]. Of note, some CLs act as embedded librarians, saving physicians time by performing literature searches and assisting in answering clinical questions that arise while rounding during real-time patient care [15, 22, 23]. A recent study suggests that deploying CLs into clinical settings in this manner could encourage physicians to answer clinical questions, become lifelong learners, and ensure patient safety [9].

Five systematic reviews have been published assessing the role of CLs in hospital systems and during rounds [15, 16, 22–24]. These reviews have concluded that CLs provide useful information to clinicians to inform their decisions. Indeed, several studies described in these reviews and elsewhere have used objective data to suggest that CLs can positively affect patients and the health care system by shortening hospital stays and reducing costs [25–28]. Other studies, described in these reviews and elsewhere, propose that CLs positively affect clinicians by adding to the culture of learning and clinical teams’ question formulation, improving clinicians’ skill in searching the literature, and facilitating the implementation of evidence-based practices [2, 28–32]. Only one existing study has objectively investigated how the presence of a CL affects clinical questioning on rounds, and it found that the presence of a CL was associated with increased trainee identification of clinical questions on rounds [33]. Other studies have been limited to self-reported surveys, and no study has investigated how the presence of a CL affects certain aspects of clinical question practices on rounds. As such, direct observation may supplement previous studies to provide further depiction of the effect of CLs on inpatient rounds with respect to clinicians’ learning and question formulation.

In this study, the authors asked whether the presence of a CL was associated with changes in inpatient teams’ clinical questioning, as measured by the number of questions asked and answered, the time spent asking and answering questions, and the quality of questions asked during rounds. Furthermore, we explored changes in inpatient team members’ self-reported ability, comfort, and confidence in searching the literature and applying evidence-based practices.

## METHODS

### Study site

University of Chicago Medicine is a 568-bed academic medical center on the South Side of Chicago. Two inpatient services were selected for participation in the study. The first service is an internal medicine inpatient team consisting of 1 to 3 medical students, 1 intern, 3 senior residents, and a hospitalist or general medicine attending physician. This service carries a maximum of 12 patients and focuses on transitioning patients from the hospital through to an outpatient setting. The second service is a general pediatric inpatient team consisting of 3 to 4 medical students, 3 interns, 2 senior residents, and a pediatric hospital medicine attending. This service usually carries fewer than 15 patients but has no upper patient limit. Both services admit patients daily.

Both an internal medicine team and a pediatric team were chosen to increase the generalizability of findings. Prior to the start of this study, the CL rounded once weekly on the internal medicine team for one year but did not round on the pediatric team. The CL had been available to any student, trainee, or staff member at the University of Chicago for consultation by phone or email, although utilization of the service was low. The CL gives an annual presentation about library services to all starting interns in their new hospital orientation.

### Rounding

A direct observation instrument (supplemental Appendix A) was developed and used during rounds to record the name of the team, the presence or absence of the CL, the number of patients seen, the total time spent rounding, the total number of clinical questions asked and answered, the time spent asking and answering clinical questions, and the content of clinical questions and nonclinical team questions about EBM resources. A clinical question was defined as a question to which the asker did not know the answer and to which a search of the medical literature might reasonably be expected to produce an answer. For example, “What are this patient’s lab results?” was not considered a clinical question, whereas “If MRSA [methicillin-resistant *Staphylococcus aureus*] is cultured in the urine of an elderly female, what further diagnostic tests or evaluations are indicated?” was considered a clinical question. The time spent asking and answering clinical questions was limited to the number of minutes spent verbalizing questions and the answers to those questions and did not include time spent looking up questions.

At the beginning of a rotation, medical students, interns, residents, and attendings were informed by a medical student observer, who had been trained by two senior researchers, that they could request a literature search by the CL in person, by email, or through an online submission form in Research Electronic Data Capture (REDCap) [34] hosted at the University of Chicago. The REDCap form asked participants to select their departments and levels of training before describing their questions (supplemental Appendix B). Participants were informed that questions submitted through REDCap would be forwarded to the CL, who would research the questions and send answers to their services’ attendings to discuss with the team within one to three days.

Participants were also given and instructed about a card outlining the basics of asking a question in the population, intervention, comparison, outcome (PICO) format [35] along with a link to library resources and the online search request form (supplemental Appendix C). The PICO information card was distributed and explained by the medical student observer at the beginning of every participant’s rotation regardless of the presence or absence of the CL.

Research of clinical questions during rounds involved the CL’s use of a tablet loaded with several medical applications and resources including UpToDate, PubMed, Micromedex, DynaMed, AccessMedicine, and Lab Tests Online. As the team discussed a patient’s care, members of the team asked the CL clinical questions, or the CL offered to research questions that arose. The CL was present before and after rounds to talk with team members regarding questions but did not interrupt presentations during rounds or provide information regarding a search, except between patient rooms. The CL occasionally solicited additional information to be able to find the most relevant results. Relevant information was summarized and explained by the CL to the team in real time. Articles and more thorough responses were emailed to the asker or the asker’s attending after rounds.

### Observation schedule

Rounding data were collected for twenty-five days when the CL was not present on rounds (“NCLR”) and for twenty-five days when the CL was present on rounds (“CLR”) (supplemental Appendix D). The medical student observer attended all fifty days of both NCLR and CLR. Since medical students, interns, residents, and attendings had different switch schedules onto and off of the teams, the observation schedule was designed to provide all groups with both CLR and NCLR.

### Analysis of question quality

All clinical questions were transcribed as stated during rounds each day for quality assessment. The most completely formed clinical question, based on PICO components, was selected by the observer at the end of rounds each day for quality assessment. Questions with more individual PICO components or with more descriptors within a given component were selected. If two questions appeared to contain the same number of PICO components and descriptors, the longer question was sent for quality evaluation. Question quality was evaluated by a blinded librarian, who was unaffiliated with the study, using criteria adapted from the rubric for the Fresno Test of Competence in Evidence-Based Medicine (supplemental Appendix E) [36], which is a reliable tool for assessing learners’ performance in clinical scenarios requiring evidence-based approaches. The rubric accompanying the Fresno Test assesses whether example clinical questions contain key PICO components and thus represent well-developed EBM questions. The rubric from the Fresno Test was validated through distribution to teachers of EBM and revised based on expert suggestions.

### Survey procedure

A 12-item post-rotation survey was developed based on review of published literature (supplemental Appendix F). Attendance was taken at the start of each day of rounds. Medical students, interns, residents, and attending participants who were present for at least one day of CLR and one day of NCLR were provided with the post-rotation survey in paper format immediately following rounds on their final days of inpatient service. Participants who were not available during that time were sent an email with an identical online REDCap survey for them to complete. Participants were asked to reflect using a Likert scale (1=very low; 5=very high) on self-perceived (1) ability to formulate a question about patient care in the PICO format, (2) comfort in conducting an online medical literature search, and (3) confidence in finding articles to answer their PICO questions before and after their rotations. Respondents were further asked about their clinical questioning during the rotation, the usefulness of having the CL on rounds, and the effect of clinical questioning on patient care. No incentive was provided for survey completion, and study involvement was not tied to learner evaluation in any way.

### Data analysis

Descriptive statistics were used to summarize variables of interest. Analysis assessed differences in clinical questioning between CLR and NCLR. Wilcoxon rank-sum tests were used for ordinal data to compare the number of questions asked and answered between CLR and NCLR and to compare the quality of questions in each PICO category and in total quality score between CLR and NCLR after grading from a blinded evaluator. Independent-sample *t*-tests were used for continuous data to compare the time spent asking or answering questions and the time spent rounding per patient between CLR and NCLR. Multivariable regression models were used to test whether the presence of the CL, department, time spent rounding per patient, academic year of residency, or participant training level were associated with question quantity or question quality. Wilcoxon signed-rank paired tests were used for paired post-rotation survey data to determine whether survey respondents’ ability, comfort, and confidence changed before and after their exposure to CL rounds. Chi-squared tests were used for categorical data to determine if survey response rates differed based on department or level of training. All analyses were performed in Stata 14.0, and statistical significance was defined as *p*<0.05 [37].

### Study authorization

This project was exempted by the University of Chicago’s Institutional Review Board (IRB16-0629).

## RESULTS

### Direct observation

Data were collected from rounds for a total of 50 days over the 10-week study period. Observations were split evenly between CLR (n=25) and NCLR (n=25) and between pediatrics (n=25) and internal medicine (n=25). The number of questions asked and answered and the time spent asking and answering clinical questions were significantly greater for CLR than for NCLR (Table 1). On 4 occasions, the CL answered questions from a previous day. In multivariable regression models controlling for department, time spent rounding per patient, and academic year of residency, our results remained unchanged.

**Table 1:** Differences in number of questions and time spent asking and answering clinical questions between clinical librarian present on rounds and clinical librarian not present on rounds

	Clinical librarian present (n=25)	Clinical librarian not present (n=25)	Test statistic	*p*-value
Number of questions asked*	5 (2–9)	3 (0–9)	−2.76	<0.01‡
Number of questions answered*	3 (1–9)	2 (0–7)	−3.35	<0.01‡
Time asking questions (min)†	1.39 (1.11)	0.52 (0.49)	−3.58	<0.01§
Time answering questions (min)†	2.15 (1.90)	1.05 (1.19)	−2.46	0.02§
Time rounding per patient (min)†	11.82 (4.30)	11.71 (4.33)	−0.09	0.93§

* Median value with range in parentheses.

† Mean value with standard deviation in parentheses.

‡ Wilcoxon rank-sum test.

§ Independent-sample *t*-test.

### Question submission

No questions were submitted through the online submission form. Five of the total 203 questions (2.5%) recorded during the study period were submitted by email to the CL. All questions posed to the CL were addressed on rounds or with a follow-up email to the asker or the asker’s attending.

### Question quality evaluation

**Figure 1:**
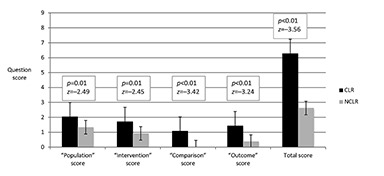
Population, intervention, comparison, outcome (PICO) question quality scores One clinical question from rounds each day (n=49) was scored using a rubric adapted from the Fresno Test by a blinded evaluator. Each question received a score from 0 to 3 in 4 categories corresponding to the elements of well-formed PICO questions. The total score was out of a maximum of 12 points.

The most complete clinical question was rated for quality from each day of rounds (n=49). There were no clinical questions asked during 1 day of rounds; therefore, no clinical question was recorded for that day. Questions from CLR were significantly more likely to contain each of the 4 components of PICO questions than questions from NCLR (Figure 1). Overall, on a scale from 0 to 12, participants scored a median of 6 (range of 1 to 12) with a mean of 6.28 (standard error of 0.71) on CLR and a median of 3 (range of 0 to 5) with a mean of 2.63 (standard error of 0.27) on NCLR. In multivariable regression models controlling for department, time spent rounding per patient, participant training level, and academic year of residency, our results remained unchanged.

### Survey findings

Fifty surveys were distributed, and 45 surveys were completed, representing a 90% response rate. In pediatrics, 28 surveys were distributed and 27 were completed (96%); while in internal medicine, 22 surveys were distributed and 18 were completed (82%). All 20 medical students completed the survey, 10 of 11 interns completed the survey (91%), 11 of 14 residents completed the survey (79%), and 4 of 5 attendings completed the survey (80%). There was no significant difference in response rate based on department (χ^2^=0.29; *p*=0.59) or level of training (χ^2^=0.48; *p*=0.92).

On a Likert scale from 1 to 5, participants reported an ability to formulate a clinical question with a median of 3 prior to their rotations and a median of 4 at the end of their rotations (*z*=−4.73; *p*<0.01). Similarly, participants reported comfort conducting a literature search with a median of 3 prior to their rotations and a median of 4 at the end of their rotations (z=−2.90; *p*<0.01). Participants reported confidence in finding an article to answer a clinical question with a median of 3 prior to their rotations and a median of 3 at the end of their rotations. However, the distribution of scores changed significantly, with 12 participants (27%) reporting a confidence level of at least 4 with respect to their pre-rotation ability and 22 participants (49%) reporting a confidence level of at least 4 with respect to their post-rotation ability (*z*=−3.60; *p*<0.01).

Participants were also asked to reflect on questions regarding their rotation experience with EBM. Thirty-three of 40 responses (83%) indicated that the CL had added to their learning, and 28 of 38 responses (74%) indicated that the CL had increased the relevance of questions they asked. Notably, several of these participants described the impact of the CL in this regard, with one writing, “it helped to get answers faster and get high quality data,” and another stating, “we learned about finding the most updated article on a topic, how to filter PubMed for reviews, and discussed how to keep up to date with reading journals.”

A third question asked how clinical questioning had changed patient care. Thirteen of 39 responses (33%) indicated that clinical questioning had changed patient care decisions. For example, a participant explained that care was changed when the CL’s research prompted them to use “Lasix [furosemide] instead of HCTZ [hydrochlorothiazide] in [a] patient with hyponatremia.” Another respondent wrote that information from the CL helped “narrow-in on a diagnosis for a patient with a complication of peritoneal dialysis.” A third participant described that research by the CL had helped them decide “whether to bridge a patient with heparin.”

### Nonclinical team questions

During CLR, there were fifteen additional nonclinical general questions directed to the CL about how to find EBM resources. These included questions about assessing articles’ sources, using PubMed effectively, and employing different search strategies for distinct types of clinical questions. These questions were not included as clinical questions in the above analysis. There were no general nonclinical questions about finding EBM resources during NCLR.

## DISCUSSION

We found that the presence of a CL on inpatient rounds was associated with increased quantity and quality of clinical questioning and was subjectively perceived as improving participants’ EBM skills and changing patient care decisions. While similar findings have been reported [15, 22, 23, 33], this study is novel in that it includes an evaluation of previously unexplored aspects of clinical questioning via direct observation of a CL’s effects on rounds. In addition, survey responses suggest that involvement of CLs in rounds can improve EBM skills and care management decisions. This supports findings reported by Marshall and colleagues, who previously described survey findings showing CLs to be perceived as valuable to clinical learning and decisions at an even higher rate than reported in this study [21].

These results are significant in that they provide evidence that the physical presence of a CL during rounds can prompt clinicians to verbalize and pursue questions, spend significantly more time discussing and answering questions, and learn about resources for EBM without significantly increasing the amount of time spent on rounds. Furthermore, the presence of a CL on rounds may be associated with changes in patient care management that are based on evidence from literature searches during rounds.

Balancing time demands with the need to practice EBM and remain up-to-date with the medical literature is a significant challenge for practicing physicians today. These stressors may contribute to physician burnout, which has been shown to have negative effects on patient care, including major medical and medication errors and suboptimal care practices [38–40]. Embedding CLs into inpatient rounds can educate providers about search strategies and provide answers to clinical questions in real-time, thus helping to reduce physicians’ daily workload and enabling them to practice high-quality evidence-based care in a more meaningful and satisfying manner.

Earlier work has reported on the obstacles preventing clinicians from answering their clinical questions [3]. This study corroborates previous findings of obstacles, given that more questions were vocalized and more answers were provided during CLR. While a consult information service may also be effective [26], the relative disuse of the online question submission form and email for information consultations in this study suggests that a consult service would not affect clinicians’ EBM practices as significantly as CLs joining rounds. Rather, this suggests that the physical presence of a CL on rounds is a more effective means of providing real-time answers to clinical questions that arise on rounds, though more work needs to be done to determine if other forms of consult information services would be more effective. Previous studies have also suggested that clinical settings may be most appropriate for learning EBM skills [41]. By offering on-site, real-time information assistance on rounds, CLs likely encourage questioning and aid the development of EBM skills and practice.

The findings from this study are significant in their implications for the potential role of CLs during rounds, though there are important limitations to note. Most significantly, the observer might have introduced bias or subjectivity into the study during the gathering of rounding data or the forwarding of the most complete clinical questions to the blinded observer. Next, this was a single-institution study, and while both pediatric and internal medicine inpatient teams were included, the results might not be broadly applicable as this institution might have a culture that cannot be applied at all academic centers. Additionally, the importance of PICO formatting has been disputed, with some authors rejecting or qualifying its usefulness and its relevance to certain types of clinical questions [5, 42–44]. We used the PICO format as a way to track changes associated with the presence of a CL, as the PICO format is relatively accepted in the literature and previous work has suggested that the PICO format improves EBM skills [44–47]. Furthermore, we did not explore long-term changes in learners’ ability to search for and implement EBM and did not directly measure patient outcomes. Likewise, self-reported abilities to search for and implement EBM were assessed only in a post-rotation survey and might be subject to recall bias. Although the surveys aimed to provide a more holistic view into EBM practices, they were distributed at the end of participants’ rotations and might not accurately reflect respondents’ abilities or the CL’s impact. Also, the extra time spent asking and answering questions on CLR might have detracted from rounds in an unmeasured way. Finally, participant behavior might have been altered on CLR by the presence of an observer. While the presence of the observer during NCLR likely controlled for this effect, the true baseline questioning habits of participants is not known.

As the role of the CL evolves, institutions that already employ CLs may benefit from embedding CLs and involving them during rounds to assist with EBM searches related to patient care. While there are many other ways to teach EBM skills, this intervention does not require extra time of already busy clinicians and functions to update clinicians’ knowledge [25–28, 48]. Future work should be done to increase the generalizability of these findings and to determine whether certain services or patients would benefit most from CL services, as has been previously suggested [12]. Furthermore, future work could investigate other ways in which CLs affect clinicians, such as by further study of changes in EBM ability and assessment of long-term maintenance of change as well as changes in provider stress and burnout.

Having a CL embedded on inpatient rounds was associated with a greater quantity and quality of clinical questions on rounds and perceived improvements in EBM skills and clinical management. As the medical literature continues to expand, clinicians must frequently update their medical knowledge despite stressful time constraints. Given the results of this study and previous work, embedding and including CLs during rounds represents a solution to improve clinicians’ EBM skills by providing real-time information, thus better connecting recent literature to clinical practice.

## SUPPLEMENTAL FILES

Appendix A Direct observation instrument

Appendix B Population, intervention, comparison, outcome (PICO) question submission form

Appendix C Population, intervention, comparison, outcome (PICO) information card

Appendix D Rounding schedule

Appendix E Adapted rubric from the Fresno Test of Competence in Evidence-Based Medicine

Appendix F Post-rotation survey
