# Supplementary material for: Evaluating the impact of clinical librarians on clinical questions during inpatient rounds
Source: J Med Libr Assoc. 2018 Apr 1;106(2):175–83. doi: 10.5195/jmla.2018.254 (PMC5886500; doi:10.5195/jmla.2018.254)
Supplement: Appendix A [file jmla-106-175-s001.pdf]

JMLA Journal of the Medical Library Association

SC

Supplemental content to
J Med Libr Assoc. Apr;106(2):dx.doi.org/10.5195/jmla.2018.254
www.jmla.mlanet.org
© Brian, Orlov, Werner, Martin, Arora, Alkureishi 2018

# Evaluating the impact of clinical librarians on clinical questions during inpatient rounds

**Riley Brian; Nicola Orlov, MD; Debra Werner, MLIS; Shannon K. Martin, MD, MS; Vineet M. Arora, MD, MAPP; Maria Alkureishi, MD, FAAP**

## APPENDIX A

### Direct observation instrument

Date: ________________________

1. Department hosting rounds
   a. Medicine
   b. Pediatrics

2. Was the clinical librarian present on rounds
   a. Yes
   b. No

3. Number of patients carried by the service ___________

4. Total time spent rounding ___________

5. Total number of patient care questions ASKED during rounds ___________

6. Total number of patient care questions ANSWERED on rounds (including questions posed on a different day) ___________

7. Time (in seconds) spent asking patient care questions ___________

8. Time (in seconds) spent answering patient care questions ___________

9. Representative questions:

   a. ________________________________________________________________

   b. ________________________________________________________________

   c. ________________________________________________________________
