# Supplementary material for: Evaluating the impact of clinical librarians on clinical questions during inpatient rounds
Source: J Med Libr Assoc. 2018 Apr 1;106(2):175–83. doi: 10.5195/jmla.2018.254 (PMC5886500; doi:10.5195/jmla.2018.254)
Supplement: Appendix B [file jmla-106-175-s002.pdf]

Journal of the Medical Library Association

Supplemental content to
J Med Libr Assoc. Apr;106(2):dx.doi.org/10.5195/jmla.2018.254
www.jmla.mlanet.org
© Brian, Orlov, Werner, Martin, Arora, Alkureishi 2018

Evaluating the impact of clinical librarians on clinical questions during inpatient rounds

Riley Brian; Nicola Orlov, MD; Debra Werner, MLIS; Shannon K. Martin, MD, MS; Vineet M. Arora, MD, MAPP; Maria Alkureishi, MD, FAAP

APPENDIX B

Population, intervention, comparison, outcome (PICO) question submission form

Please do NOT include any patient identifiers in your submission.

1. Service
   - Pediatrics
   - Medicine

2. Level of training
   - MS3
   - Sub-I
   - Intern
   - Resident
   - Attending

3. PICO question
   ____
   ____
   ____
   ____
