# Supplementary material for: Evaluating the impact of clinical librarians on clinical questions during inpatient rounds
Source: J Med Libr Assoc. 2018 Apr 1;106(2):175–83. doi: 10.5195/jmla.2018.254 (PMC5886500; doi:10.5195/jmla.2018.254)
Supplement: Appendix D [file jmla-106-175-s004.pdf]

JMLA Journal of the Medical Library Association

Supplemental content to
J Med Libr Assoc. Apr;106(2):dx.doi.org/10.5195/jmla.2018.254
www.jmla.mlanet.org
© Brian, Orlov, Werner, Martin, Arora, Alkureishi 2018

Evaluating the impact of clinical librarians on clinical questions during inpatient rounds

Riley Brian; Nicola Orlov, MD; Debra Werner, MLIS; Shannon K. Martin, MD, MS; Vineet M. Arora, MD, MAPP; Maria Alkureishi, MD, FAAP

APPENDIX D

Rounding schedule

June 2016

Sunday	Monday	Tuesday	Wednesday	Thursday	Friday	Saturday
			1	2	3	4
5	6	7	8	9	10	11
12	13 PEDS CL ROUNDING	14 MEDICINE CL ROUNDING	15 PEDS	16 MEDICINE CL ROUNDING	17 PEDS CL ROUNDING	18
19	20 PEDS	21 MEDICINE	22 PEDS	23 MEDICINE	24 MEDICINE	25
26 *Start of new academic year*	27 PEDS	28 MEDICINE	29 PEDS	30 MEDICINE		

JMLA Journal of the Medical Library Association

SC
Supplemental content to
J Med Libr Assoc. Apr;106(2):dx.doi.org/10.5195/jmla.2018.254
www.jmla.mlanet.org
© Brian, Orlov, Werner, Martin, Arora, Alkureishi 2018

July 2016

Sunday	Monday	Tuesday	Wednesday	Thursday	Friday	Saturday
					1<br>MEDICINE	2
3	4<br>HOLIDAY	5<br>MEDICINE<br>CL ROUNDING	6<br>PEDS<br>CL ROUNDING	7<br>MEDICINE<br>CL ROUNDING	8<br>PEDS<br>CL ROUNDING	9<br>PEDS
10	11<br>PEDS	12<br>MEDICINE<br>CL ROUNDING	13<br>PEDS	14<br>MEDICINE<br>CL ROUNDING	15<br>MEDICINE<br>CL ROUNDING	16
17	18<br>PEDS<br>CL ROUNDING	19<br>MEDICINE<br>CL ROUNDING	20<br>PEDS<br>CL ROUNDING	21<br>MEDICINE	22<br>MEDICINE	23
24	25<br>PEDS	26<br>MEDICINE	27<br>PEDS	28<br>MEDICINE	29<br>PEDS	30

JMLA Journal of the Medical Library Association

Supplemental content to
J Med Libr Assoc. Apr;106(2):dx.doi.org/10.5195/jmla.2018.254
www.jmla.mlanet.org
© Brian, Orlov, Werner, Martin, Arora, Alkureishi 2018

August 2016

Sunday	Monday	Tuesday	Wednesday	Thursday	Friday	Saturday
31	1 PEDS CL ROUNDING	2 MEDICINE CL ROUNDING	3 PEDS CL ROUNDING	4 MEDICINE CL ROUNDING	5 MEDICINE CL ROUNDING	6
7	8 PEDS CL ROUNDING	9 MEDICINE CL ROUNDING	10 PEDS CL ROUNDING	11 MEDICINE CL ROUNDING	12 PEDS CL ROUNDING	13
14	15 PEDS CL ROUNDING	16 MEDICINE	17 PEDS	18 MEDICINE	19 PEDS	20
21	22	23	24	25	26	27
28	29	30	31			
