# Supplementary material for: Evaluating the impact of clinical librarians on clinical questions during inpatient rounds
Source: J Med Libr Assoc. 2018 Apr 1;106(2):175–83. doi: 10.5195/jmla.2018.254 (PMC5886500; doi:10.5195/jmla.2018.254)
Supplement: Appendix E [file jmla-106-175-s005.pdf]

Journal of the Medical Library Association

Supplemental content to
J Med Libr Assoc. Apr;106(2):dx.doi.org/10.5195/jmla.2018.254
www.jmla.mlanet.org
© Brian, Orlov, Werner, Martin, Arora, Alkureishi 2018

Evaluating the impact of clinical librarians on clinical questions during inpatient rounds

Riley Brian; Nicola Orlov, MD; Debra Werner, MLIS; Shannon K. Martin, MD, MS; Vineet M. Arora, MD, MAPP; Maria Alkureishi, MD, FAAP

APPENDIX E

Adapted rubric from the Fresno Test of Competence in Evidence-Based Medicine

	**Population**	**Intervention**	**Comparison**	**Outcome**
**Excellent** (3 pts)	Multiple relevant descriptors	Specific intervention of interest	Specific alternative of interest	Outcome that is objective and meaningful to patient
**Strong** (2 pts)	One appropriate descriptor	Type of intervention	Specific comparison group	Nonspecific outcome
**Limited** (1 pt)	General descriptor	Mentions intervention	Mentions comparison	Reference to outcome
**Not evident** (0 pts)	Not evident	Not evident	Not evident	Not evident

Example questions with grading:

The question "What is the recommended duration of therapy for a urinary tract infection (UTI) in a neonate or infants?" received 2 points for population (neonates or infants with a UTI) and no points for other categories for a total of 2 points.

The question "Does duloxetine cause constipation in adults?" received 1 point for population (adults), 3 points for intervention (duloxetine), no points for comparison, and 2 points for outcome (constipation) for a total of 6 points.

The question "In the fluid management of adult sickle cell patients, what is the effect of using hypotonic fluids vs. isotonic fluids on inducing hypernatremia?" received 3 points for population (adult sickle cell patients in need of fluid management), 3 points for intervention (hypotonic fluids), 3 points for comparison (isotonic fluids), and 3 points for outcome (hypernatremia) for a total of 12 points.
