# Supplementary material for: Evaluating the impact of clinical librarians on clinical questions during inpatient rounds
Source: J Med Libr Assoc. 2018 Apr 1;106(2):175–83. doi: 10.5195/jmla.2018.254 (PMC5886500; doi:10.5195/jmla.2018.254)
Supplement: Appendix F [file jmla-106-175-s006.pdf]

JMLA Journal of the Medical Library Association

SC

Supplemental content to
J Med Libr Assoc. Apr;106(2):dx.doi.org/10.5195/jmla.2018.254
www.jmla.mlanet.org
© Brian, Orlov, Werner, Martin, Arora, Alkureishi 2018

Evaluating the impact of clinical librarians on clinical questions during inpatient rounds

Riley Brian; Nicola Orlov, MD; Debra Werner, MLIS; Shannon K. Martin, MD, MS; Vineet M. Arora, MD, MAPP; Maria Alkureishi, MD, FAAP

APPENDIX F

Post-rotation survey

Please circle your level of training:				
MS3	Sub-I	Intern	Resident	Attending

If applicable, please circle your department:			
Medicine	Pediatrics	Med/Peds	Other:

Please circle the number that corresponds to your response:

PRIOR to this rotation on Maroon/Medicine, please answer the following questions in relation to your skills:	Very low	Low	Moderate	High	Very high
Ability to formulate a question about patient care in the PICO format	1	2	3	4	5
Comfort conducting an online medical literature search	1	2	3	4	5
Confidence in finding an article that answers a PICO question	1	2	3	4	5

Now that you have completed your Maroon/Medicine rotation, please answer the following questions in relation to your skills:	Very low	Low	Moderate	High	Very high
Ability to formulate a question about patient care in the PICO format	1	2	3	4	5
Comfort conducting an online medical literature search	1	2	3	4	5
Confidence in finding an article that answers a PICO question	1	2	3	4	5

Did you have a chance to ask and present a clinical question related to patient care ON ROUNDS during your rotation?

____

Can you think of a time that it changed patient care? If so, please describe in a sentence or two

____

Did having the medical librarian present on rounds help you ask more relevant questions?

____

Did having the medical librarian present on rounds add to your learning?

____
